# Lifestyle changes after retirement: a Grounded Theory

**DOI:** 10.1590/0034-7167-2024-0387

**Published:** 2025-06-20

**Authors:** Sonia Silva Marcon, Verônica Francisqueti Marquete, Thiago Privado da Silva, Mayckel da Silva Barreto, Maria do Carmo Fernandez Lourenço Haddad, Helena Maria Almeida Macedo Loureiro, Vitoria Vasconcelos Logullo, Sueli Mutsumi Tsukuda Ichisato

**Affiliations:** I Universidade Estadual de Maringá. Maringá, Paraná, Brazil; II Universidade Estadual do Paraná. Paranavaí, Paraná, Brazil; III Universidade Federal do Rio de Janeiro. Rio de Janeiro, Rio de Janeiro, Brazil; IV Universidade de Aveiro, Escola Superior de Saúde. Aveiro, Portugal

**Keywords:** Grounded Theory, Retirement, Quality of Life, Worker Health, University Professor., Jubilación, Investigación Cualitativa, Estilo de Vida, Autocuidado, Interacción Social.

## Abstract

**Objective:**

to understand the changes that occur in professors’ lifestyle after retirement with regard to self-care.

**Methods:**

qualitative research that used Symbolic Interactionism as a theoretical framework and Grounded Theory, a constructivist approach, as a methodological framework. Data were collected in 2022, through remote interviews with 18 professors from a public university in northwestern Paraná.

**Results:**

with retirement, professors have greater availability and flexibility in their schedules, favoring positive changes in lifestyle, with greater appreciation of self-care actions, such as physical exercise and healthy eating, taking care of appearance, stimulating memory, a more active social life and adopting hobbies, expressed in the category “Enjoying retirement and taking up new activities”.

**Final considerations:**

retired professors, influenced by their social interactions with friends, family, healthcare professionals and co-workers throughout their lives, at this particular stage, tend to value self-care more.

## RESUMEN

**Objetivo:**

comprender los cambios ocurridos en el estilo de vida luego del retiro de profesores universitarios en términos de autocuidado.

**Métodos:**

investigación cualitativa que utilizó como marco teórico el Interaccionismo Simbólico y, como marco metodológico, la Teoría Fundamentada, de vertiente constructivista. Los datos fueron recolectados en 2022, a través de entrevistas remotas a 18 profesores de una universidad pública del noroeste de Paraná.

**Resultados:**

con la jubilación los docentes tienen mayor disponibilidad y flexibilidad en sus horarios, favoreciendo cambios positivos en el estilo de vida, con mayor valorización de acciones de autocuidado, como ejercicio físico y alimentación saludable, cuidado de la apariencia, estimulación de la memoria, vida social más activa y adopción de hobbies, expresados en la categoría “Disfrutar del retiro y realizar nuevas actividades”.

**Consideraciones finales:**

los docentes jubilados, debido a la influencia de sus interacciones sociales con amigos, familiares, profesionales de la salud y compañeros de trabajo a lo largo de su vida, en esta etapa en particular, tienden a valorar más el autocuidado.

## INTRODUCTION

Population aging and longevity are increasing worldwide. The Brazilian population is aging very rapidly. Estimates suggest that one in four Brazilians will be over 65 in 2060, and a considerable portion will be retired^(1)^.

Retirement is a period of transition in which various phenomena of biological, psycho-emotional, social and ecological change occur, making this life event a specific and unique experience throughout the human life cycle, affecting not only its leading actor, but the entire microsystem in which it is inserted - its family^(2)^. This is particularly important when one considers that, very often, retirement is marked by aging, a stage in the life cycle in which changes in aerobic function, balance, flexibility and muscular strength can occur, directly reflecting on individuals’ cognitive, work and functional capacities^(3)^.

Lifestyle is understood as a complex system formed by several elements that are related to each other and that change and are modified in the same health context^(4)^. It plays a major role in individuals’ conditions and quality of life, especially in old age. Thus, the main risk factors for illness due to chronic non-communicable diseases are linked to the population’s lifestyle, such as smoking, alcohol consumption, unhealthy diet and physical inactivity. These factors can be influenced by changes in behavior and by government actions that regulate and reduce, for instance, the marketing, consumption and exposure of products that are harmful to health^(5)^.

Changing lifestyle to achieve better health and quality of life is a challenge at any stage of the life cycle^(6)^, especially in old age, when habits are more ingrained and the context in which older adults find themselves prevents decision-making and the implementation of changes. However, retirement can represent a valuable opportunity to implement changes that are often desired but unfeasible given the daily demands of the job market.

The purpose of adopting a healthier lifestyle is translated into the performance of self-care behaviors^(7,8)^, which involve the ability, willingness and actions in relation to autonomy and management of one’s own health. Self-care itself is established as the ability of a person, family and community to develop attitudes aimed at self-care in the physical, emotional, social and mental spheres. Its actions encompass health promotion and preservation, prevention, control and coping with diseases and disabilities, with or without the help/guidance of a healthcare professional^(9)^.

Historically, self-care has been interpreted as an alternative to the clinical-medical care model, but over time, its understanding has expanded to a multidimensional approach integrated into the holistic conception of health^(10)^. In the field of nursing, this notion gained greater prominence after the formulation of the Self-Care Deficit Theory, developed by Dorothea E. Orem, which emphasized the centrality of the human factor in self-care. In general, self-care can help prevent health problems, stabilize and control symptoms, preserve bodily functionality, and promote self-management capacity, based on self-determined and informed choices. To achieve these goals, it is necessary to take into account both individual aspects and systemic dynamics^(11)^.

The absence or deficiency of good self-care practices by older adults triggers an excessive search for health resources, social assistance and medical care, increasing the likelihood of hospitalizations and health expenses. Older adults who do not practice self-care tend to have more and varied mental disorders, while those who are committed to their psychological and physical health are more likely to maintain emotional independence, have a better perception of their quality of life and greater control over negative responses in life^(12)^.

Individuals’ social interaction with friends and family influences the way they think, act and make decisions and, through interactions, symbols and meanings, is shared and understood. In other words, individuals will indicate to others what they should do and will interpret indications made by others. Thus, human action is based on what they point out, interpret and assess^(13)^, and this influences the way in which self-care is exercised.

The literature points to disagreement regarding the implications of retirement on people’s lives. This is related to the fact that the process of retiring constitutes a complex, personified phenomenon, with meanings attributed by multiple possible interpretations in a dynamic world. In order to better capture this entire context, qualitative research would be needed^(14)^. However, in practice, it is observed that many studies that address the topic are based on the positivist paradigm and the quantitative methodological approach^(15-17)^. Thus, the study in question, based on qualitative research, proposes to analyze in depth the changes that occur in lifestyle after retirement with regard to self-care.

## OBJECTIVE

To understand the changes that occur in professors’ lifestyle after retirement regarding self-care.

## METHODS

### Ethical aspects

The research followed the ethical precepts of Resolution 466/2012 and the Guidelines for Procedures in Research with Any Stage in a Virtual Environment (Brazilian National Research Ethics Committee/2021), and the project was approved by the Research Ethics Committee of the signatory institution (Opinion 4,316,457/2020). All participants expressed their agreement to participate in the study by signing the Informed Consent Form, which was sent via messaging application and returned via the same means. To ensure participant anonymity, excerpts of their statements were identified by means of codes composed of the letter “P” (retired professor), followed by an Arabic numeral indicating the order in which the interview was conducted and the identification of the sample group to which they belong, followed by information on the time since retirement (e.g., P1G1, 30 months of retirement).

### Study design and theoretical-methodological framework

This is a qualitative and explanatory study that used Symbolic Interactionism (SI) as a theoretical framework and Grounded Theory (GT) as a methodological framework. The criteria established in the COnsolidated criteria for REporting Qualitative research (COREQ)^(18)^ were used as a support tool to write the study report.

SI is a theoretical perspective centered on human interaction, as it advocates that people act based on the meanings that things have for them. These meanings, in turn, are generated and modified from social interactions, with an emphasis on subjective interpretation in the construction of social reality^(12)^. This framework shapes all stages of data analysis and interpretation of results. GT, in turn, which has conceptual bases in SI, aims to allow the construction of a theoretical model that facilitates the understanding of social phenomena from the perspective of the subjects investigated^(19)^.

In this study, in relation to the methodological framework, the constructivist approach of GT^(19)^ was adopted, which values the researcher’s reflexivity on their own influences and preconceptions in the research process, in addition to recognizing that data and theory are co-constructed by the researcher and participants. This approach seeks to understand participants’ subjective meanings and experiences, considering the social and historical aspect of the context in which participants find themselves^(19)^.

### Study setting

The research was conducted at a public university located in the northwest region of the state of Paraná, which has six regional *campi* and offers more than 70 on-site undergraduate courses. The university does not provide its employees with a formal retirement preparation program. In accordance with current legislation, employees (technicians and professors) who have already met the minimum requirements for retirement and chose to continue working were granted the right to receive the retention bonus^(20)^. Professors linked to graduate studies may request, subject to approval by the relevant body and the establishment of a contract with the university, to become volunteer professors. In this way, they may supervise master’s, doctoral and scientific initiation courses, in addition to offering courses in graduate studies and coordinating/participating in research/extension projects. However, they may not hold any institutional position.

### Methodological procedures

*A priori*, it was defined that any professor retired by age or length of service, regardless of the length of retirement or area of activity, could participate in the study. The only exclusion criterion previously defined was not having access to the internet and/or a device that would allow the interview to be conducted remotely, due to restrictions imposed by the pandemic period at the time, and the greater fragility of older adults.

The retirees were invited to participate in the study by telephone contact from the main researcher, at which time she introduced herself and the study proposal as well as explaining how she had obtained the professor’s contact and the type of participation desired. In general, all those contacted demonstrated empathy with the study proposal and felt flattered to have been nominated/selected. Only four of those contacted did not agree to participate in the study.

Data were collected between August 2021 and June 2022 through individual interviews conducted via video call using the Google Meet^®^ app, which were previously scheduled and held on a day and time that was most convenient for participants. They were audio-recorded after authorization and lasted an average of one hour and ten minutes. All interviews and their transcriptions were conducted by the same researcher (a doctoral student in nursing with experience in collecting and analyzing qualitative data), who had no relationship with participants.

At the beginning of the interviews, participants were asked for information about their age, retirement time, qualifications, area of activity, activities related to the area of training they were currently undertaking and family income.

The interviews were directed according to the hypotheses that emerged in the sample groups, with a guiding question being used in all interviews: “What changes occurred in your lifestyle after retirement?”. This fact was identified during the intensive interview and interpreted through the SI theoretical framework, which made it possible to understand the changes that occurred in lifestyle after retirement, translated by self-care in relation to health.

In total, 18 retired professors participated in the study, distributed into three sample groups. The first of these was defined intentionally and consisted of eight professors who were assigned to the Health Sciences Center, three of whom were indicated by one of the researchers, who did not participate in data collection.

As the data were collected and analyzed, hypotheses emerged that led to interviews being conducted in new sample groups, taking into account the analytical resource of theoretical sampling proposed by the method. Thus, the first sample group showed that healthcare professionals were very attached to or tired of their work, due to their greater concern with direct care for human beings, and that this even led them to anticipate their retirement. Thus, the following questions emerged: do professors from other areas also change their retirement plans due to concern for the health of others? In what situations does this occur? These questions, in turn, led to the formulation of the following hypothesis: the intentionality of healthcare professors in retiring is mediated by professional fatigue and also by concern for the health of close family members rather than their own, and this does not occur with professors from other areas.

Thus, the second sample group consisted of five professors from other areas (exact sciences, humanities and biological sciences). The data obtained from this sample group indicated that, regardless of their area of activity, four of them did not aspire to retirement and had even postponed it. Thus, the following hypothesis arose: the continuity of professional activity is linked to professors’ attachment and social status, and this may negatively affect the retirement experience.

The third sample group consisted of retirees who continued to work as volunteer professors in graduate courses or who had started a new activity related to their field of study. The following questions were asked to the five professors in this group: what motivates you to continue working? What was your relationship with work like before retirement? What was your meaning of life before retirement? And what is that meaning now?

The number of participants was limited by the simultaneous data collection and analysis. This process was completed with the third sample group, at which point theoretical saturation was reached, when it was perceived that the conceptual categories developed were well developed in terms of their properties and dimensions, as recommended by the research method in question.

### Data analysis

Data collection, analysis and categorization occurred simultaneously, through constant comparative data analysis and use, throughout the research process, of analytical tools, such as memos and diagrams^(19)^. In the memos, insights and hypotheses raised by the researcher were recorded, and the diagrams constituted alternative visual mechanisms for the concretization of ideas, scope, direction and existing connections between the categories^(19)^.

The initial coding and analysis process was carried out using MAXQDA^®^ Plus 2020 Student software and occurred in two interdependent stages: initial and focused coding. In initial coding, the data were analyzed word by word (*in vivo* code) and/or line by line and/or incident by incident, resulting in provisional codes. In other words, the process began with data collection during the interviews. These data were initially coded, identifying and naming text fragments that seemed relevant, without following predefined categories. This coding helped to capture the nuances and different aspects of the participants’ experiences. As new data were collected, constant comparison between the initial codes and the new data was carried out. This allowed for the refinement and adjustment of the codes, as well as the identification of patterns, similarities and differences^(20)^.

In focused coding, separation, classification, synthesis/integration and organization were carried out by surveying the most significant and/or frequent codes identified in the previous stage. Based on the emerging similarities between codes, they were grouped into broader categories. Subcategories emerged when variations or more specific aspects were identified within a main category. This process enabled the construction of subcategories and categories, which, in turn, helped to identify the central phenomenon of the research^(20)^.

In the constructivist model, categories and subcategories are constructed in interaction with the data. This means that interpretations were influenced by their context and by the researcher’s interactions with participants, playing a central role in defining these subcategories^(20)^.

As the subcategories were identified, we sought to understand how they related to each other and to the main categories. The aim was to develop a theory that explains the experience or phenomenon studied in a rich and detailed manner, allowing a deeper understanding of the meaning of the social interactions involved^(20)^.

The data analysis stages were interpreted in light of SI. In this context, the identification of categories and concepts emerging from the data collected was directly influenced by social interactions and the meanings attributed by participants. Through constant comparison of the data, we sought to understand how individuals construct their realities based on the changes that occurred after retirement, with social interaction, and their self in the process of self-reflection. The analysis, therefore, valued both the social construction of meaning and the symbolic interaction among participants.

At the end of the data analysis process, a substantive theory was constructed called “Experiencing changes in retirement mediated by interactions with the social and family context and the singularities of the aging process”, which is made up of three categories. This paper presents the category “Enjoying retirement and carrying out new activities”.

## RESULTS

The 18 retired professors studied were between 57 and 77 years old, 11 were female, 15 were married, two were divorced and one was single. The length of time since retirement varied between one and 20 years. All had a doctoral degree, including two post-doctoral degrees. Seven were from the nursing department, three from biology, two from history, two from psychology, and the others from the mathematics, literature, pharmacy and pedagogy departments. Five were still volunteering in graduate studies; two occasionally participated in master’s/doctoral defense committees or in the issuance of opinions for scientific journals; two had started a new career related to their field of study; and the other five were not engaged in any professional activity. Family income varied from ten to 30 minimum wages.

**Figure 1:**
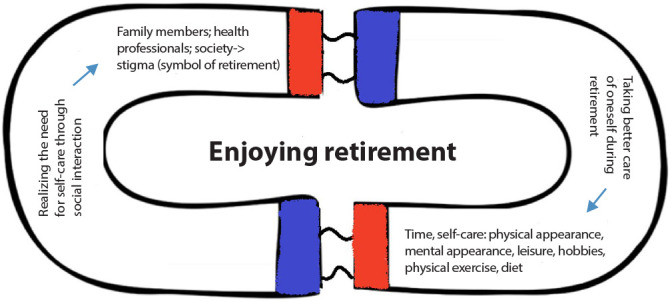
Diagram of the category “Enjoying retirement” and its subcategories. Maringá, Paraná, Brazil, 2024

In Figure 1, it is possible to observe how the subcategories, constructed through an interactive and interpretative process, in which the researcher and the data influence each other, relate to each other, highlighting the importance of social interaction in carrying out activities aimed at self-care in health. Analogous to the representation of a magnet, it is observed that the experience of a person during retirement has two poles. Initially, there is a greater propensity for a person to suffer from fear of stigmatization that retirement brings to social coexistence and interpretation. Once this initial difficulty is overcome, retirees begin to recognize that there are benefits that could not be experienced previously, mainly resulting from greater availability and flexibility for time management. This, in turn, encourages them to envision possibilities of adopting more consistent and constant self-care practices, leading to “enjoying retirement”.

### Realizing the need for self-care through social interaction

In the interaction between retired professors and healthcare professionals, the latter provided guidance on the importance of self-care, while family members expressed concern about comorbidities resulting from the aging process. This greater contact with family members tends to lead to direct changes in the actions that retirees will adopt in terms of caring for their health.

The symbol representing retirement, arbitrarily established by a portion of society through social interaction, was also found to be something negative and of great social stigma. It is possible to see in the excerpts this social interaction that occurs daily:

*Every time I go to the Geriatrician, she asks, “So, have you started exercising?”. Then I go to the cardiologist and he says, “So, are you exercising?”* [laughs]. *There is no way around it, we have to do it* [...] (P24G3, 18 months of retirement)

*I feel like they* [the children] *take greater care of me. They get worried. Sometimes they want me to take a language course, I don’t know what, “Oh, you have to read, you have to do different things.” My son, especially, is very much like that, you know, I think he’s afraid that I’ll become senile, with Alzheimer’s.* (P11G1, six years of retirement)

*After a certain age, we start to worry about stimulating the brain* [...] *because, with age, we start to have some synapse deficiencies. So, learning a language, a musical instrument, having a social life, singing, dancing or doing some activity that stimulates dopamine, serotonin, is very important. You have to have constant stimulation, because otherwise we really end up with many memory deficiencies.* [...] (P1G1, 30 months of retirement)

*What is the social idea that people have of retirees, that old woman who doesn’t know how to put on makeup* [...] *who gets in the way* [...] (P21G2, six years of retirement)

Furthermore, the stigmatized symbol was pointed out by some participants regarding retirement as the final stage of a life cycle and aging as the end of life. This symbol is developed through social interaction, in which retirees, when interpreting each other’s actions, carry out self-reflection and have a response according to the meaning they attribute to these actions. This response will be their action in the way they will experience retirement:

*I think it is an extremely important phase, because it will be the last phase of your life* [...]. *So, may it be worth it. May it be lived with joy, with health, with all the possibilities it gives you* [...]. *She gives you a lot of chances, but you have to see it.* (P13G2, 15 years of retirement)

*The pulmonologist said, “Take care of your parents, you brought them here to live, not to die.” And that phrase stayed with me - I have to live, I didn’t retire to die.* [...] *the image we have in Brazil of retirees is that they will die soon. I didn’t want to be treated this way* (P21G2, six years of retirement)

*I haven’t retired from myself and my relationship with the world. I’m still active, it’s a “fake” retirement* [laughs]. *I’m not that retiree who put on his pajamas* [laughs] *and is walking around the house like a zombie. No, that’s not it! I’m HORRIFIED when people say I’m retired* [laughs] [...] *retirement no longer has that aura of old age, of the end of the line; back then, it could have been true.* (P10G3, two years of retirement)

Social interaction between retired professors, healthcare professionals and family members highlights the importance of self-care and influences the way retirement is experienced and interpreted.

### Taking better care of oneself during retirement

One of the major changes that occurs in retirement is the greater availability of time, which allows retirees to prioritize themselves, to focus on themselves, to self-knowledge, a fact that conditions their lifestyle:

[...] *It’s about having more time for myself*, [...] *when we’re working, we take a back seat* [...]. *So, I think that taking care of you, I think it’s the inversion, so I’m a priority now, it’s not my job.* (P11G1, six years of retirement)

[...] *and also live, think with myself, be with myself. So, it’s been really good to be with myself* [...] *feeling that self-knowledge, the search for knowing myself was an important path for me was very important* [...] *that now I live more in my house, more with myself.* (P5G1, three years of retirement)

*The most important lesson of retirement was learning to know myself better. I didn’t have much time to get to know myself properly during my life, given the pressure I had on perfectionism, so it allowed me to get to know myself better* [...] (P14G2, three years of retirement)

The reflective and interpretative process, both consciously and unconsciously, and the lack of desire to reproduce the stigmatized symbol of retirement result in greater self-care, through the practice of physical exercise and/or care with appearance, memory, diet, social life and hobbies:

*Retirement is associated with a quiet life. If I take care of my health, I won’t let this happen to me* [...] *I’m no longer living a youth, I’m already old age. So, I have the responsibility to take care of myself, my health, my diet, my body. I think this is my obligation.* (P21G2, six years of retirement)

Regarding physical appearance, self-care is about deconstructing the idea that older adults do not know how to put on makeup and take care of themselves. In this way, they adopt a new approach of taking better care of themselves:

*One thing I decided to do was stop dyeing my hair. Embrace my age. I dyed my hair until about five years ago, but I decided to let the gray show and I’m happy.* [...] *I have hair that’s compatible with my age, but there are women who are 45 and already have gray hair like mine and are embracing it, I think it’s beautiful. Imagine, I dyed it until I was 60* (laughs). (P24G3, 18 months of retirement)

*I’m going to a hairdresser that I like more. It’s true that it’s a little more expensive, but I have to do it for myself* [...]. *If I think it’s prettier, if I like it, why not do it for myself? And there’s also one thing: if I don’t do it now, when will I? It has to be done, so do it for yourself.* (P21G2, six years of retirement)

During retirement, changes occur in social and family life, and this can be perceived with greater attention, tranquility and strengthening of bonds. At this stage, new values and priorities are established, such as family and friends:

*Look, it’s a different life, now I live more in my house, more with myself, with my family, with my friends here on the street. It’s really a new life.* (P5G1, three years of retirement)

*I was always much more dedicated to work than to my family, and then things changed. Today I am very dedicated to my family. After retiring for a while, my focus shifted to my family. I stopped doing other things. My family is my daily life.* (P14G2, three years of retirement)

*I also have some close friends, who we support each other. I’ve traveled a few times, and before, even if I traveled, I would be like: I have to give a test, I have to correct a test, I have to prepare a program, I have to release the grades, I mean, those work tasks, they no longer exist.* (P21G2, six years of retirement)

Physical healthcare occurs more frequently, even when a person still works voluntarily, as there is greater flexibility in schedules:

*I continue doing what I like, without the daily obligations. For instance, I don’t have to leave home at seven thirty, seven o’clock, to get to university. I leave later, whenever I want, and I walk, calmly, I got used to walking, because it’s good for me* [...] (P8G2, five years of retirement)

*When I worked, I didn’t go to the gym because I didn’t have time. For me, I had to wake up and go to university, not go to a gym and then go to university. After I got off work, I was tired and didn’t want to go to the gym.* (P14G2, three years of retirement)

*I started to worry more about my health, to seek medical or laboratory assistance to better assess my physical condition, and then we can organize ourselves in this sense of self-care.* (P1G1, 27 months of retirement)

Greater flexibility in schedules also allows for the adoption of care related to food:

*While you’re working, you have less time to eat, and you do everything in a rush. You sit down to have lunch and dinner, but it’s always a bit more rushed, and with retirement, that’s over.* (P13G2, 15 years of retirement)

*I think that, in fact, the diet ends up being healthier. We end up having more time to prepare healthier meals. When you’re in a rush, you always want the quickest, easiest thing to do. Now, I can eat healthier, eating lots of vegetables, fruits, and salads.* [...] (P4G2, 30 months of retirement)

Finally, in retirement, people have the opportunity to carry out activities that are considered pleasurable and always desired, but are unfeasible due to the lack of compatible schedules or, then, carried out with less intensity/frequency:

*Retirement is also an opportunity, not a necessity, to start another profession/activity. It is the opportunity to do other things, to do manual work, to make music.* (P9G3, two years of retirement)

*I had little time for leisure-related activities* [...] *so there was a big difference in that sense, of having more time to do the things I like, that give me pleasure, that I even did, but in a restricted way* [...] (P2G1, three years of retirement)

*I really like washing cars. The cars are always clean,* [...] *both mine and my son’s.* (P14G2, three years of retirement)

*I think crafts are a source of wealth, they entertain. They develop creativity and skill, so I really like them. Oh, I made a lot of little coats for my grandchildren. Every now and then I make something to give as a gift.* (P24G3, 18 months of retirement)

*I sing in a group for older adults* [...] *it’s something that is very rewarding. It fills your soul when you sing* [...] *I create song lyrics* [...] *it’s something that completes me. I really like singing* [...] (P14G2, three years of retirement)

In retirement, greater availability of time allows individuals to prioritize self-care, strengthen social and family ties, adopt healthy habits and carry out pleasurable activities that were previously unfeasible.

## DISCUSSION

The findings of this study show that, although retirement is still understood as representing the finitude of human beings, it is also understood positively as an opportunity to enjoy life better. Thus, retired professors, consciously or unconsciously, to a greater or lesser extent, experience positive changes in relation to self-care, which is the result of a self-reflective process and social interaction with family members, healthcare professionals, coworkers and the often stigmatized symbol of what it means to be retired.

From the results, it was possible to see that retirement provided/enabled more time to dedicate to self-care actions, in addition to greater self-awareness and self-knowledge. Research carried out with 5,034 Dutch individuals, aged between 60 and 65 years, identified that retirement exposes people to new social contexts, and this can lead them to reassess their orientations and ways of living^(21)^.

Thus, through the self-nomination process, retirees determine their line of action, the lifestyle they intend to have and the role they will take in society. In other words, self-reflection is the central mechanism by which retirees will begin to face and deal with the world.

Thus, aging and retirement can be considered as one of the objects that, through the self, trigger changes in self-care, such as greater stimulation of memory, healthier eating, physical exercise and regular consultations with healthcare professionals. In the case of retired professors, these behaviors may result from social interaction with people who also have greater access to knowledge about the importance of these attitudes for health, and this theme may be involved in different interactions (workshops, conferences, participation in panels, ad hoc opinions).

Furthermore, the fact that retirement is more often associated with aging and has a stigmatized symbol can trigger changes in the behavior of retirees, through greater involvement in self-care. Hence, they hope not to be remembered as that person who has outlived their usefulness to the family and/or community, does not contribute to social development and is only a burden. Interaction with other people allows for self-reflection and, consequently, for rethinking and modifying, or not, conduct, attitudes, behaviors and life plans.

The desire, as a retiree, not to be seen in a stigmatized way arises from the formation of the object of oneself, through the process of role playing. The way in which individuals see themselves, how others see or define them, or even the way in which a person sees themselves taking over a role can vary. In other words, during their experience, a person may have interacted with other retirees who adopted them as a model to be followed or rejected, shaping their conduct^(13)^.

The importance of social interaction for older adults was reaffirmed in a study conducted with 12 participants from a social group, who pointed to ageism as a factor that hinders the social integration and interaction of older adults. The data also showed that older adults’ quality of life is closely related to healthy eating, physical exercise, family relationships, spirituality and religiosity^(22)^.

The greater willingness to perform self-care actions, which is often neglected during the period of professional activity, in addition to a process of social interaction and interpretation, also involves greater flexibility and availability of schedules. A qualitative study, carried out with 20 nurses from the basic and hospital network, in the state of Rio de Janeiro, highlighted how much lifestyle is compromised and determined by work, because, often, personal care, such as social life, pleasures, needs and activities directed at self-care, are neglected, due to numerous work commitments. The greater focus that many people give to work is related to social status and remuneration^(23)^.

The appreciation and flexibility of working hours in retirement were also highlighted in a systematic review of longitudinal studies. In this study, it was possible to identify that greater autonomy to manage free time was associated with an increase in the time spent practicing sports, leisure and physical activity^(17)^.

In the same vein, a study conducted in Brazil, which used the Longitudinal Study of Health of Older Adults in Brazil as a basis, with a sample of 9,412 individuals aged 50 or over, found that greater availability of time enabled and encouraged individuals to invest more in their health. It even led them to perceive it as less laborious to go to a doctor’s appointment or cook healthier foods^(24)^.

A study conducted in Japan, using data obtained from the Japan Household Panel Survey with individuals aged 50 or over, retired and non-retired, found that, on average, retirees were more satisfied with leisure activities, health and life than non-retired individuals. Furthermore, retirement was associated with psychological well-being, with a decline in psychosomatic and somatic symptoms, psychological distress, with improvements in life satisfaction, as well as increases in physical exercise habits^(25)^.

In Brazil, data from the National Household Sample Survey indicate that retirement has given family members greater availability for leisure, healthcare and well-being, providing benefits for personal and family life, resulting in a decrease in the occurrence of various diseases and in health indicators as a whole. In other words, retirement provides more time to take care of oneself and those who are part of one’s daily life^(26)^.

The benefits of self-care in retirement include increased leisure time, greater flexibility in working hours, and the absence of daily commitments. A study of 5,405 Americans aged 50 and over found that activities such as reading, crafts, and music tend to stimulate and activate memory, attenuating the negative association between retirement and decline in cognitive function and depressive symptoms^(27)^.

In this context, Swedish research investigated the longitudinal association between leisure activity and depressive symptoms in the first three years of retirement in 1,033 retirees, finding that those with intense depressive symptoms at the beginning of the study had linear reductions in involvement in social/leisure activities. Individuals who had greater involvement in social and physical activities at the beginning of retirement reported a reduction in depressive symptoms^(28)^. Therefore, the importance of preparing for retirement is highlighted so that people can develop strategies to mitigate the repercussions on mental health. In any case, healthcare professionals need to be alert to identify signs of depression early and provide healthcare according to the needs presented, considering the comprehensiveness of care.

It is worth remembering that the self-care deficit in older adults is related to a higher prevalence of comorbidities, a deterioration in the subjective perception of health, an increasing dependence on performing activities of daily living, and a greater propensity for regular physical inactivity, which exacerbates functional decline. Therefore, the development of strategic and targeted interventions that consider the specificities of aging and promote the integration of individuals into public policies and community resources is essential. These actions aim to foster autonomy and strengthen the practice of self-care, contributing to improving quality of life and reducing negative impacts on the health system^(29)^.

Therefore, self-care plays a crucial role in maintaining the physical, emotional, mental, spiritual and social health of individuals. Regularly assessing needs in each of these areas, identifying dysfunctional self-care patterns and obstacles to their implementation enables a more individualized approach, favoring the adoption of appropriate and conscious practices and behaviors^(30)^.

It is important to note that activities undertaken during retirement are shaped by positive or negative perceptions about the aging process. In this sense, a study conducted with 24 high-performance athletes who had been retired for more than four years observed that those with positive ideas about aging felt motivated to practice physical exercise, while those who had a negative perception, marked by fears, insecurities and stigmas, associated athletic retirement with losses. Preparation for retirement and choosing the time when it should occur influenced its acceptance and, consequently, behaviors after this event. Furthermore, interaction with family members proved to be of unique importance, by encouraging and guiding self-care practices, such as, for example, staying physically active^(31)^.

The role of interaction with family members was also highlighted in a study conducted in China, which found the importance of companionship and care from spouses in maintaining a healthy lifestyle, especially for older adults over 70 years of age. It was also found that the negative impact of living alone on eating habits was greater for women than for men, probably due to differences in social status and family responsibilities. The less social identity a person has, the more negative impact they will suffer when they lose one of them, and after retirement, men tend to maintain many social identities, while women tend to assume the role of housewife^(32)^.

In this regard, a study conducted in Germany with 11,168 people aged 45 to 80 years highlights that healthcare professionals should consider, in a holistic manner, the behavioral factors and circumstances in which retired people find themselves. It is not enough to simply encourage the practice of physical exercise and leisure, due to this greater flexibility of schedules in retirement and senescence, but a partnership should be established and a jointly developed care plan (professional and retiree) should be developed that is individualized, considering possible obstacles to exercise, such as joint problems, muscle weakness, availability of structural and social resources for this activity, among others^(33)^.

Another aspect to be considered is that taking care of one’s appearance is related to the rules that exist in the social environment, and can be a reflection of social interactions, freedom, self-knowledge, self-determination and recognition^(34)^. However, such care does not always provide benefits to the mental and physical health of individuals, because it may be performed only to avoid stigmas arising from negative symbols associated with aging and not because of an expressed personal desire. In this sense, it is reiterated that carrying out self-care activities motivated only to avoid stigmas is not beneficial.

It is essential to understand how retirees interact with the world and with their own selves, because sometimes people even develop satisfactory self-care actions, but internally these actions may have more to do with the stigma of being retired, rather than with the desire to fully enjoy the benefits that retirement can bring. This is why the results of the benefits and harms of retirement in people’s lives are so divergent^(35)^.

Therefore, strategies are needed to ensure that retirement occurs in a personalized and satisfactory manner. It is suggested that people prepare for retirement by participating in interventions with interdisciplinary approaches, through consultations with specialized professionals from different areas, instructional lectures and group meetings^(2,36)^.

### Study limitations

Possible limitations of this study may be related to the fact that the interviews were conducted remotely due to restrictions imposed by the COVID-19 pandemic. This may have prevented people with difficulty accessing the remote network from participating in the study. Furthermore, the fact that participants were included based on peer recommendations and their willingness to share their experiences after retirement may have limited the results.

However, despite the theoretical risk of bias due to the predominance of a specific area (nursing), the empirical data did not show a considerable impact of this difference, as the desire or even need for changes was also perceived by healthcare professionals, demonstrating that training and professional practice are not sufficient to guarantee the adoption of healthier lifestyles before retirement, which strengthens the reliability of the study.

### Contributions to health and nursing

The results of the study provide important contributions to a better understanding of the implications of retirement on retiree’s lifestyle. Therefore, understanding, from the perspective of retirees themselves, the meanings involved in adopting new health-related behaviors that result in self-care measures enhances the possibilities of planning actions, at an individual and collective level, aimed at improving the living conditions and health of retirees.

The results also highlight the importance of healthcare professionals valuing the characteristics of the different stages of the human life cycle, recognizing that those in pre-retirement and also retirees need a broader perspective so that they can constitute a source of support and encouragement for behavioral changes, especially those related to well-being and health in this important stage of life.

## FINAL CONSIDERATIONS

It was identified that the experience of a person during retirement has two poles. Initially, there is a greater tendency for the person to suffer from the fear of the stigmatization that retirement brings to social life and social interpretation. Once this initial difficulty is overcome, retirees begin to recognize that there are benefits that could not be experienced previously, mainly due to greater availability and flexibility in time management. This, in turn, encourages them to envision possibilities of adopting more consistent and constant self-care practices. There is a greater dedication of time to practicing hobbies and physical exercise, in addition to taking care of appearance, memory, diet and social life. This occurs due to the influence of social interactions (with friends, family, healthcare professionals, coworkers) throughout life and, at this stage in particular, due to greater availability and flexibility of schedules and as a result of the aging process itself.
