# Supplementary material for: Lifestyle changes after retirement: a Grounded Theory
Source: Rev Bras Enferm. 2025 Jun 20;78(2):e20240387. doi: 10.1590/0034-7167-2024-0387 (PMC12185146; doi:10.1590/0034-7167-2024-0387)
Supplement: Translation

# Translation: Mudanças no estilo de vida após a aposentadoria: uma teoria fundamentada

**DOI:** 10.1590/0034-7167-2024-0387pt

**Authors:** Sonia Silva Marcon, Verônica Francisqueti Marquete, Thiago Privado da Silva, Mayckel da Silva Barreto, Maria do Carmo Fernandez Lourenço Haddad, Helena Maria Almeida Macedo Loureiro, Vitoria Vasconcelos Logullo, Sueli Mutsumi Tsukuda Ichisato

**Affiliations:** I Universidade Estadual de Maringá. Maringá, Paraná, Brasil; II Universidade Estadual do Paraná. Paranavaí, Paraná, Brasil; III Universidade Federal do Rio de Janeiro. Rio de Janeiro, Rio de Janeiro, Brasil; IV Universidade de Aveiro, Escola Superior de Saúde. Aveiro, Portugal

**Keywords:** Aposentadoria, Pesquisa Qualitativa, Estilo de Vida, Autocuidado, Interação Social.

## Abstract

**Objetivo:**

compreender as mudanças ocorridas no estilo de vida após a aposentadoria de professores universitários no tocante ao autocuidado.

**Métodos:**

pesquisa qualitativa que utilizou como referencial teórico o Interacionismo Simbólico e, metodológico, a Teoria Fundamentada nos Dados, vertente construtivista. Os dados foram coletados em 2022, mediante entrevistas remotas com 18 professores de universidade pública do noroeste do Paraná.

**Resultados:**

com a aposentadoria, professores têm maior disponibilidade e flexibilidade de horários, favorecendo mudanças positivas no estilo de vida, com maior valorização de ações de autocuidado, como prática de exercícios físicos e alimentação saudável, cuidados com aparência, estimulação da memória, vida social mais ativa e adoção de hobbies, expressas na categoria “Desfrutando da aposentadoria e realizando novas atividades”.

**Considerações finais:**

professores aposentados, por influência de suas interações sociais com amigos, familiares, profissionais de saúde e colegas de trabalho ao longo da vida, nesta etapa em particular, tendem a valorizar mais o autocuidado.

## INTRODUÇÃO

O envelhecimento populacional e a longevidade se apresentam de forma crescente em todo o mundo. A população brasileira está envelhecendo muito rapidamente. Estimativas sugerem que um em cada quatro brasileiros terá mais de 65 anos em 2060, e uma parcela considerável será de aposentados^(1)^.

A aposentadoria é um período de transição no qual ocorrem variados fenômenos de mudança biológica, psicoemocional, social e ecológica que fazem deste acontecimento de vida uma experiência específica e única ao longo do ciclo vital humano, afetando não apenas o seu protagonista, mas todo o microssistema em que se insere - a sua família^(2)^. Isso é particularmente importante quando se considera que, muito frequentemente, a aposentadoria é demarcada pelo envelhecimento, etapa do ciclo vital em que podem ocorrer alterações na função aeróbica, equilíbrio, flexibilidade e força muscular, refletindo diretamente nas capacidades cognitiva, laboral e funcional do indivíduo^(3)^.

O estilo de vida é entendido como um sistema complexo formado por diversos elementos que se relacionam entre si e que se modifica e é modificado em um mesmo contexto de saúde^(4)^. Ele tem papel preponderante nas condições e qualidade de vida do indivíduo, sobretudo na velhice. Destarte, os principais fatores de risco para o adoecimento por doenças crônicas não transmissíveis estão interligados ao estilo de vida da população, como tabagismo, consumo de álcool, alimentação não saudável e inatividade física. Estes fatores podem ser influenciados pela mudança de comportamento e por ações governamentais que regulamentem e reduzam, por exemplo, comercialização, consumo e exposição de produtos danosos à saúde^(5)^.

Modificar o estilo de vida para alcançar um melhor estado de saúde e qualidade de vida constitui desafio em qualquer etapa do ciclo vital^(6)^, especialmente na velhice, quando os hábitos estão mais enraizados e o contexto em que o idoso se encontra impede a tomada de decisão e a concretização de mudanças. Contudo, a aposentadoria pode representar uma oportunidade valiosa para implementar mudanças que muitas vezes são desejadas, mas inviáveis diante das demandas cotidianas quando se está inserido no mercado de trabalho.

O propósito de adotar um estilo de vida mais saudável é traduzido pela realização de comportamentos de autocuidado^(7,8)^, os quais envolvem a capacidade, a disposição e as ações em relação à autonomia e ao gerenciamento da própria saúde. O autocuidado em si é estabelecido como a aptidão da pessoa, família e comunidades para desenvolver atitudes voltadas para o cuidado de si nos âmbitos físico, emocional, social e mental. Suas ações englobam a promoção e preservação da saúde, prevenção, controle e enfrentamento de doenças e incapacidades, com ou sem ajuda/orientação de um profissional de saúde^(9)^.

Historicamente, o autocuidado foi interpretado como uma alternativa ao modelo de cuidado clínico-médico, mas, com o tempo, sua compreensão se expandiu para uma abordagem multidimensional integrada à concepção holística de saúde^(10)^. No campo da enfermagem, essa noção ganhou maior destaque a partir da formulação da Teoria do Déficit de Autocuidado, desenvolvida por Dorothea E. Orem, que colocou ênfase na centralidade do fator humano no cuidado de si. De maneira geral, o autocuidado pode auxiliar na prevenção de agravos à saúde, na estabilização e controle de sintomas, na preservação da funcionalidade corporal e na promoção da capacidade de autogerenciamento, com base em escolhas autodeterminadas e informadas. Para alcançar tais propósitos, é necessário que sejam levados em consideração tanto os aspectos individuais quanto as dinâmicas sistêmicas^(11)^.

A ausência ou deficiência de boas práticas de autocuidado por pessoas idosas desencadeia, de maneira excessiva, a busca por recursos de saúde, assistência social e cuidados médicos, aumentando a probabilidade de hospitalizações e gastos com a saúde. Idosos que não realizam o autocuidado tendem a ter mais e variados transtornos mentais, enquanto os engajados com a sua saúde psicológica e física têm maiores chances de manter a independência emocional, ter melhor percepção sobre sua qualidade de vida e maior controle sobre as respostas negativas da vida^(12)^.

A interação social do indivíduo com amigos e familiares influencia a forma como ele pensa, age e toma decisões e, por intermédio das interações, símbolos e significados, é compartilhada e compreendida. Ou seja, o indivíduo indicará ao outro o que deve realizar e interpretará indicações realizadas pelos outros. Desse modo, a ação humana tem como base o que ele aponta, interpreta e avalia^(13)^, e isso influencia a forma de exercitar o autocuidado.

A literatura aponta discordância em relação às implicações da aposentadoria na vida das pessoas. Isso se relaciona ao fato de o processo do aposentar-se constituir um fenômeno complexo, personificado, com significados atribuídos por múltiplas possíveis interpretações em um mundo dinâmico. Todo esse contexto, para ser melhor captado, precisaria do emprego de pesquisas qualitativas^(14)^. Contudo, na prática, observa-se que muitos estudos que abordam a temática estão fundamentados no paradigma positivista e na abordagem metodológica quantitativa^(15-17)^. Assim, o estudo em tela, a partir de pesquisa qualitativa, propõe-se a analisar de maneira aprofundada as mudanças ocorridas no estilo de vida após a aposentaria no tocante ao autocuidado.

## OBJETIVO

Compreender as mudanças ocorridas no estilo de vida após a aposentadoria de professores universitários no tocante ao autocuidado.

## MÉTODOS

### Aspectos éticos

No desenvolvimento da pesquisa, foram seguidos os preceitos éticos da Resolução nº 466/2012 e as Orientações para Procedimentos em Pesquisas com Qualquer Etapa em Ambiente Virtual (Comissão Nacional de Ética em Pesquisa/2021), e seu projeto foi aprovado pelo Comitê de Ética em Pesquisa com Seres humanos da instituição signatária. Todos os participantes manifestaram anuência em participar do estudo assinando o Termo de Consentimento Livre e Esclarecido, o qual foi enviado por aplicativo de mensagem e devolvido pela mesma via. Para assegurar o anonimato dos participantes, os extratos de suas falas foram identificados por meio de códigos compostos pela letra “P” (professor aposentado), seguida de número arábico indicativo da ordem de realização da entrevista e a identificação do grupo amostral a que pertence, seguido da informação sobre o tempo de aposentadoria (ex: P1G1, 30 meses de aposentadoria).

### Tipo de estudo e referencial teórico-metodológico

Estudo de abordagem qualitativa e caráter explicativo, que utilizou como referencial teórico o Interacionismo Simbólico (IS) e, metodológico, a Teoria Fundamentada nos Dados (TFD). Para a redação do relatório do estudo, foram utilizados critérios estabelecidos no *COnsolidated criteria for REporting Qualitative research* (COREQ)^(18)^ como ferramenta de apoio.

O IS é uma perspectiva teórica centrada na interação humana, pois preconiza que as pessoas agem com base nos significados que as coisas têm para elas. Estes significados, por sua vez, são gerados e modificados a partir das interações sociais, com valorização da interpretação subjetiva na construção da realidade social^(12)^. Esse referencial molda todas as etapas da análise dos dados e interpretação dos resultados. A TFD, por sua vez, que possui bases conceituais no IS, tem como propósito permitir a construção de um modelo teórico que facilite o entendimento dos fenômenos sociais a partir da perspectiva dos sujeitos investigados^(19)^.

No presente estudo, em relação ao referencial metodológico, foi adotada a vertente construtivista da TFD^(19)^, que valoriza a reflexividade do pesquisador sobre suas próprias influências e preconcepções no processo de pesquisa, além de reconhecer que os dados e a teoria são co-construídos pelo pesquisador e pelos participantes. Esta abordagem busca compreender os significados subjetivos e as experiências dos participantes, considerando o aspecto social e histórico do contexto onde os participantes se encontram^(19)^.

### Cenário do estudo

A pesquisa foi realizada em universidade pública localizada na região Noroeste do estado do Paraná, que possui seis *campi* regionais e oferta mais de 70 cursos de graduação presenciais. A universidade não disponibiliza para os servidores um programa formal de preparação para a aposentadoria. Em conformidade com a legislação vigente, aos servidores (técnicos e docentes) que já cumpriram os requisitos mínimos para a aposentadoria e optaram por continuar no exercício da função, foi concedido o direito de usufruir do abono de permanência^(20)^. Os docentes vinculados à pós-graduação podem solicitar, mediante aprovação do órgão de interesse e estabelecimento de um contrato com a universidade, sua condição como professor voluntário. Deste modo, pode orientar mestrado, doutorado e iniciação científica, além de ofertar disciplinas na pós-graduação e coordenar/participar de projetos de pesquisa/extensão. Contudo, não pode ocupar qualquer cargo institucional.

### Procedimentos metodológicos

*A priori*, definiu-se que qualquer professor aposentado por idade ou tempo de serviço, independentemente do tempo de aposentadoria ou da área de atuação, poderia participar do estudo. O único critério de exclusão previamente definido foi não ter acesso à rede de internet e/ou dispositivo que possibilitasse a realização da entrevista de forma remota, devido às restrições impostas pelo período pandêmico na ocasião, e a condição de maior fragilidade de pessoas idosas.

Os aposentados foram convidados a participar do estudo mediante contato telefônico da pesquisadora principal, ocasião em que se apresentava e a proposta do estudo, bem com esclarecia sobre como conseguiu o contato do professor e o tipo de participação desejada. De uma forma geral, todos os contactados demonstraram empatia com a proposta do estudo e se sentiram lisonjeados em terem sido indicados/selecionados. Apenas quatro dos contactados não aceitaram participar do estudo.

Os dados foram coletados entre agosto de 2021 e junho de 2022, mediante entrevistas individuais realizadas por vídeo chamada no aplicativo *Google Meet* ^®^, as quais foram previamente agendadas e realizadas em dia e horário mais conveniente ao participante. Elas foram audiogravadas após autorização, e tiveram duração média de uma hora e dez minutos. Todas as entrevistas e suas transcrições foram realizadas pela mesma pesquisadora (doutoranda em enfermagem com experiência em coleta e análise de dados qualitativos), que não tinha qualquer tipo de relação com os participantes.

No início das entrevistas, foram solicitadas aos participantes informações sobre idade, tempo de aposentadoria, titulação, área de atuação, atividades relacionadas à área de formação que realizava atualmente e renda familiar.

As entrevistas foram direcionadas de acordo com as hipóteses surgidas nos grupos amostrais, sendo utilizada uma pergunta norteadora em todas as entrevistas: “Quais mudanças ocorreram em seu estilo de vida após a aposentadoria?”. Tal fato foi identificado durante a entrevista intensiva e interpretado por intermédio do referencial teórico do IS, o qual possibilitou compreender as mudanças ocorridas no estilo de vida após a aposentadoria, traduzidas pelo autocuidado em relação à saúde.

Ao todo, participaram do estudo 18 professores aposentados, distribuídos em três grupos amostrais. O primeiro deles foi definido de forma intencional, e foi constituído por oito docentes que eram lotados no Centro de Ciências da Saúde, três dos quais foram indicados por uma das pesquisadoras, a qual não participou da coleta de dados.

À medida que os dados eram coletados e analisados, foram surgindo hipóteses que direcionaram à realização de entrevistas em novos grupos amostrais, em consideração ao recurso analítico da amostragem teórica proposto pelo método. Assim sendo, o primeiro grupo amostral evidenciou que os profissionais da área da saúde tinham muito apego ou cansaço do trabalho, devido a maior preocupação no cuidado direto do ser humano, e que isso os levava inclusive a antecipar a aposentadoria. Emergiram, assim, os seguintes questionamentos: será que professores de outras áreas também alteram o planejamento de sua aposentadoria em decorrência da preocupação com a saúde de outrem? Em que situações isto ocorre? Estes questionamentos, por sua vez, levaram à formulação da seguinte hipótese: a intencionalidade dos professores da área da saúde em se aposentar é mediada pelo cansaço profissional e também pela preocupação com a saúde de familiares próximos do que com a sua própria, e isso não ocorre com professores de outras áreas.

Desse modo, o segundo grupo amostral foi constituído por cinco professores de outras áreas (exatas, humanas e ciências biológicas). Os dados obtidos neste grupo amostral apontaram que, independentemente da área de atuação, quatro deles não aspiravam a aposentadoria e, inclusive, a postergaram. Assim, surgiu a seguinte hipótese: a continuidade do exercício da atividade profissional está ligada ao apego e *status* social de professor, e isso pode afetar negativamente a experiência da aposentadoria.

Porquanto, o terceiro grupo amostral foi composto por aposentados que continuavam trabalhando como professores voluntários na pós-graduação ou que iniciaram uma nova atividade relacionada à sua área de formação. Junto aos cinco professores deste grupo, foram utilizadas as seguintes questões: o que te motiva a continuar desempenhando atividades laborais? Como era a sua relação com o trabalho antes da aposentadoria? Qual era o seu sentido de viver antes de se aposentar? E qual é esse sentido agora?

O número de participantes foi delimitado pela coleta e análise concomitante dos dados. Esse processo foi finalizado com o terceiro grupo amostral, momento em que foi alcançada a saturação teórica, quando se percebeu que as categorias conceituais elaboradas estavam bem desenvolvidas no tocante às suas propriedades e dimensões, conforme preconiza o método de pesquisa em tela.

### Análise dos dados

A coleta, análise e categorização dos dados ocorreram de forma simultânea, por meio da análise comparativa constante dos dados e utilização, durante todo o processo de pesquisa, de ferramentas analíticas, como memorandos e diagramas^(19)^. Nos memorandos, foram registrados *insights* e hipóteses levantadas pela pesquisadora, e os diagramas constituíram mecanismos visuais alternativos à concretização de ideias, alcance, direção e conexões existentes entre as categorias^(19)^.

O processo inicial de codificação e análise foi realizado no *software MAXQDA* ^®^ *Plus 2020 Student* e ocorreu em duas etapas interdependentes: codificação inicial e focalizada. Na codificação inicial, os dados foram analisados palavra por palavra (código *in vivo*) e/ou linha a linha e/ou incidente por incidente, emergindo códigos provisórios. Ou seja, o processo começou com a coleta dos dados durante as entrevistas. Esses dados foram codificados inicialmente, tendo-se identificado e nomeado fragmentos de texto que pareciam relevantes, sem seguir categorias predefinidas. Essa codificação ajudou a captar as nuances e diferentes aspectos das experiências dos participantes. À medida que novos dados foram coletados, realizou-se a comparação constante entre os códigos iniciais e os novos dados. Isso permitiu refinar e ajustar os códigos, além de identificar padrões, similaridades e diferenças^(20)^.

Já na codificação focalizada, realizaram-se a separação, classificação, sintetização/integração e organização com levantamento dos códigos mais significativos e/ou frequentes identificados na etapa anterior. Com base nas similaridades emergentes entre os códigos, eles foram agrupados em categorias mais amplas. As subcategorias surgiram quando se identificaram variações ou aspectos mais específicos dentro de uma categoria principal. Esse processo possibilitou a construção de subcategorias e categorias, as quais, por sua vez, auxiliaram na identificação do fenômeno central da pesquisa^(20)^.

No modelo construtivista, as categorias e subcategorias são construídas em interação com os dados. Isso significa que as interpretações foram influenciadas pelo seu contexto e pelas interações da pesquisadora com os participantes, desempenhando um papel central na definição dessas subcategorias^(20)^.

À medida que as subcategorias foram identificadas, buscou-se entender como elas se relacionavam entre si e com as categorias principais. O objetivo foi desenvolver uma teoria que explique a experiência ou o fenômeno estudado de maneira rica e detalhada, permitindo uma compreensão mais profunda do significado das interações sociais envolvidas^(20)^.

As etapas de análise dos dados foram interpretadas à luz do IS. Neste contexto, a identificação de categorias e conceitos emergentes a partir dos dados coletados foi diretamente influenciada pelas interações sociais e pelos significados atribuídos pelos participantes. Por intermédio da comparação constante dos dados, buscou-se compreender como os indivíduos constroem suas realidades a partir das mudanças ocorridas a partir da aposentadoria, com a interação social, e seu *self* no processo de autorreflexão. A análise, portanto, valorizou tanto a construção social do significado quanto a interação simbólica entre os participantes.

No final do processo de análise dos dados, construiu-se a teoria substantiva nominada “Vivenciando mudanças na aposentadoria mediadas por interações com o contexto social e familiar e pelas singularidades do processo de envelhecimento”, a qual é constituída por três categorias. Nesta comunicação, é apresentada a categoria “Desfrutando da aposentadoria e realizando novas atividades”.

## RESULTADOS

Os 18 docentes aposentados em estudo tinham entre 57 e 77 anos, sendo 11 do sexo feminino, 15 casados, dois divorciados e um solteiro. O tempo de aposentadoria variou entre um ano e 20 anos. Todos tinham o título de doutor, sendo dois pós-doutores. Sete eram do departamento de enfermagem, três, da biologia, dois, da história, dois, da psicologia, e os demais, dos departamentos de matemática, letras, farmácia e pedagogia. Cinco ainda estavam atuando como voluntários na pós-graduação; dois participavam eventualmente em bancas de defesa de mestrado/doutorado ou na emissão de pareceres para revistas científicas; dois iniciaram uma nova carreira relacionada à sua área de formação; e os outros cinco não realizavam qualquer atividade profissional. A renda familiar variou de dez a 30 salários mínimos.

Na Figura 1, é possível observar como as subcategorias, construídas por meio de um processo interativo e interpretativo, em que o pesquisador e os dados se influenciam mutuamente, se relacionam entre si, destacando a importância da interação social na realização de atividades voltadas para o autocuidado em saúde. Analogamente à representação de um imã, observa-se que a experiência da pessoa durante a aposentadoria possui dois pólos. Inicialmente, há uma maior propensão a que a pessoa sofra com o medo da estigmatização que o aposentar-se traz para o convívio e a interpretação social. Uma vez superada essa dificuldade inicial, os aposentados passam a reconhecer que há benefícios que anteriormente não podiam ser experienciados, decorrentes principalmente de maior disponibilidade e maleabilidade para a gestão do tempo. Isso, por sua vez, os impulsiona a vislumbrar possibilidades de adoção de práticas mais consistentes e constantes de autocuidado, levando ao “desfrutando da aposentadoria”.

**Figura 1:** Diagrama da categoria “Desfrutando da aposentadoria” e suas subcategorias. Maringá, Paraná, Brasil, 2024

### Percebendo a necessidade do autocuidado por meio da interação social

Na interação dos docentes aposentados com os profissionais de saúde, estes orientavam sobre a importância do autocuidado, enquanto os familiares manifestaram preocupação quanto às comorbidades decorrentes do processo de envelhecimento. Esse maior contato com os familiares tende a proporcionar mudanças diretas nas ações que os aposentados adotarão diante do cuidado com a sua saúde.

Também se verificou o símbolo que representa a aposentadoria, estabelecido arbitrariamente por uma parcela da sociedade, por meio da interação social, como algo negativo e de grande estigma social. É possível verificar nos excertos essa interação social que ocorre cotidianamente:

*Cada vez que eu vou na Geriatra, ela pergunta: “E aí, começou a fazer exercício?”. Aí eu vou no cardiologista e ele fala: “E aí, está fazendo exercício?”* [risos]. *Não tem jeito de escapar, a gente tem que fazer* [...]. (P24G3, 18 meses de aposentadoria)

*Eu sinto assim que eles* [filhos] *têm um cuidado maior comigo. Eles ficam preocupados. Às vezes, querem que eu faça um curso de línguas, não sei o quê: “AI, você tem que ler, você tem que fazer coisas diferentes”. Meu filho, principalmente, ele é muito assim, sabe, acho que ele tem medo que eu fique senil, com Alzheimer.* (P11G1, seis anos de aposentadoria)

*Depois de uma certa idade, a gente vai tendo essa preocupação de estimular o cérebro* [...] *porque, com a idade, a gente vai ficando com algumas deficiências de sinapses. Então, aprender um idioma, um instrumento musical, ter uma vida social, cantar, dançar ou fazer alguma atividade que estimule as dopaminas, as serotoninas, é muito importante. Você tem que ter estímulos constantes, porque senão a gente vai ficando realmente com muitas deficiências de memória* [...] (P1G1, 30 meses de aposentadoria)

*Que é a ideia social que as pessoas têm dos aposentados, aquela velha que não sabe se maquiar* [...] *que atrapalha* [...] (P21G2, seis anos de aposentadoria)

Além do mais, o símbolo estigmatizado foi apontado por alguns participantes sobre a aposentadoria como etapa de finalização de um ciclo de vida e, o envelhecimento, como a finitude da vida. Esse símbolo é desenvolvido por meio da interação social, no qual os aposentados, ao interpretarem as ações uns dos outros, realizam uma autorreflexão e têm uma resposta conforme o significado que eles atribuem a essas ações. Essa resposta será a sua ação da forma como vivenciará a aposentadoria:

*Acho que é uma fase extremamente importante., porque vai ser a última fase da sua vida* [...]. *Então, que ela valha a pena. Que seja vivida com alegria, com saúde, com toda possibilidade que ela lhe dá* [...]. *Ela lhe dá muitas chances, mas você tem que enxergar.* (P13G2, 15 anos de aposentadoria)

*O médico pneumologista falou: “Cuide dos seus pais, você os trouxe para cá para viver, não para morrer”. E essa frase ficou comigo - tenho que viver, não me aposentei para morrer.* [...] *a imagem que nós temos no Brasil de aposentado é que ele vai morrer logo. Eu não queria ser tratada desta forma.* (P21G2, seis anos de aposentadoria)

*Eu não me aposentei comigo mesmo e na minha relação com o mundo. Continuo na ativa, é uma aposentadoria “fake”* [risos]. *Eu não sou aquele aposentado que colocou o pijama* [risos] *e está aí andando pela casa feito um zumbi. Não, não é isso! Eu tenho HORROR que digam que eu sou aposentado* [risos] [...] *aposentadoria não tem mais essa áurea de velhice, de fim de linha; lá atrás, podia ser verdade.* (P10G3, dois anos de aposentadoria)

A interação social entre docentes aposentados, profissionais de saúde e familiares ressalta a importância do autocuidado e influencia a forma como a aposentadoria é vivenciada e interpretada.

### Cuidando mais de si mesmo durante a aposentadoria

Uma das grandes mudanças que ocorre na aposentadoria é a maior disponibilidade de tempo, o que permite aos aposentados priorizar a si mesmo, direcionar o olhar para si, para um autoconhecimento, fato que condiciona o seu estilo de vida:

[...] *é eu ter tempo mais para mim,* [...] *quando a gente está trabalhando, a gente fica em segundo plano* [...]. *Então, eu acho que esse cuidar do ti, acho que é a inversão, então eu sou prioridade agora, não é o meu trabalho.* (P11G1, seis anos de aposentadoria)

[...] *e viver também, pensar comigo mesmo, ficar comigo. Então, está sendo muito bom estar comigo* [...] *sentir que o autoconhecimento, a busca por se conhecer era um caminho importante para mim foi muito importante* [...] *que agora eu vivo mais na minha casa, mais comigo.* (P5G1, três anos de aposentadoria)

*A lição mais importante da aposentadoria foi aprender a me conhecer melhor. Eu não tive muito tempo de me conhecer direito durante a vida, dada à cobrança que eu tinha do perfeccionismo, então permitiu me conhecer* [...] (P14G2, três anos de aposentadoria)

O processo reflexivo e interpretativo, tanto consciente quanto inconscientemente, e o não querer reproduzir o símbolo estigmatizado da aposentadoria resultam em um maior autocuidado, mediante a prática de exercícios físicos e/ou cuidados com a aparência, memória, alimentação, vida social e *hobbies:*

*A aposentadoria é associada a uma vida pacata. Se eu cuidar da minha saúde, não deixo que isso aconteça para mim* [...] *não é mais uma juventude que estou vivendo, já é uma velhice. Então, tenho a responsabilidade de me cuidar, cuidar da saúde, da alimentação, do corpo. Acho que isso é uma obrigação minha.* (P21G2, seis anos de aposentadoria)

Referente à aparência física, o autocuidado é no sentido de desconstruir a ideia de que os idosos não sabem se maquiar e se cuidar. Dessa forma, adotam uma nova conduta de se cuidar mais:

*Uma coisa que eu resolvi fazer foi parar de pintar o cabelo. Assumir a minha idade. Pintei os cabelos até uns cinco anos atrás, mas resolvi deixar o grisalho aparecer e estou feliz.* [...] *eu tenho cabelo compatível com a minha idade, mas tem mulheres que com 45 anos que já está com o cabelo grisalho igual ao meu e estão assumindo, acho lindo. Imagina, eu pintei até os 60 anos* [risos]. (P24G3, 18 meses de aposentadoria)

*Estou indo em um cabeleireiro que me agrada mais. Está certo que é um pouco mais caro, mas tenho que fazer por mim* [...]. *Se acho mais bonito, se gosto, por que não fazer para mim? E também tem uma coisa, se eu não fizer agora, quando que eu vou fazer? Tem que ser feito, então faça por você.* (P21G2, seis anos de aposentadoria)

Durante a aposentadoria, ocorrem mudanças na vivência social e familiar, e isso pode ser percebido com maior atenção, tranquilidade e fortalecimento dos vínculos. Nessa fase, novos valores e prioridades são estabelecidos, como a família e amigos:

*Olha, é uma vida diferente, agora vivo mais na minha casa, mais comigo, com minha família, com os amigos aqui da rua. É uma nova vida mesmo.* (P5G1, três anos de aposentadoria)

*Sempre fui muito mais dedicado ao trabalho do que à família, e depois a coisa se inverte, hoje sou muito dedicado à família. Depois de um tempo de aposentado, meus olhos se voltaram mais para a família. Deixei de fazer outras coisas, minha família é o meu dia a dia.* (P14G2, três anos de aposentadoria)

*Tenho também alguns amigos mais próximos, que a gente se dá um apoio. Fiz algumas viagens, coisa que antes, mesmo que eu viajasse, ficava: tenho que aplicar prova, tenho que corrigir prova, tem que preparar tal programa, tem que lançar as notas, quer dizer, aquelas tarefas do trabalho, elas deixaram de existir.* (P21G2, seis anos de aposentadoria)

O cuidado com a saúde física ocorre com maior frequência, mesmo quando a pessoa ainda atua de forma voluntária, pois existe maior flexibilidade de horários:

*Continuo fazendo o que gosto, sem aquelas obrigações do dia a dia. Por exemplo, não preciso sair de casa sete e meia, sete horas, para chegar na universidade. Saio mais tarde, a hora que eu quiser, e vou a pé, tranquilo, acostumei andar, porque me faz bem* [...] (P8G2, cinco anos de aposentadoria)

*Quando trabalhava, não fazia academia, porque não tinha tempo. Para mim, eu tinha que acordar e estar com o pé dentro da universidade, e não em uma academia e depois universidade. Depois que saía do serviço, estava cansado, não queria saber de academia*. (P14G2, três anos de aposentadoria)

*Comecei a me preocupar mais com minha saúde, a procurar assistência médica, ou laboratorial, para avaliar melhor a minha condição física, e aí a gente consegue se organizar nesse sentido de autocuidado.* (P1G1, 27 meses de aposentadoria)

A maior maleabilidade de horários também permite a adoção de cuidados relacionados à alimentação:

*Enquanto você está trabalhando, você tem um tempo menor para alimentação, faz tudo meio corrido. Senta para almoçar, jantar, mas é sempre mais apressado, e com a aposentadoria, isso acabou.* (P13G2, 15 anos de aposentadoria)

*Eu acho que, na verdade, a alimentação acaba sendo mais saudável. A gente acaba tendo mais tempo para preparar comidas mais saudáveis. Quando está naquela pressa, sempre quer o mais rápido, mais fácil de fazer. Agora, consigo fazer uma alimentação mais saudável, como muito legumes, frutas, saladas* [...] (P4G2, 30 meses de aposentadoria)

Enfim, na aposentadoria, as pessoas têm a oportunidade de realizar atividades consideradas prazerosas e sempre almejadas, mas inviáveis devido à indisponibilidade de horários compatíveis ou, então, realizadas em menor intensidade/frequência:

*A aposentadoria também é a oportunidade, e não uma necessidade, de iniciar uma outra profissão/atividade. É a oportunidade de fazer outras coisas, de fazer trabalhos manuais, de fazer música.* (P9G3, dois anos de aposentadoria)

*Eu tinha pouco tempo para atividades relacionadas à área de lazer* [...] *então, houve uma grande diferença nesse sentido, de ter mais tempo para fazer as coisas que gosto, que me dão prazer, que eu até fazia, mas de forma restrita* [...] (P2G1, três anos de aposentadoria)

*Gosto muito de lavar carro. Os carros estão sempre limpos,* [...] *tanto o meu quanto o do meu filho.* (P14G2, três anos de aposentadoria)

*Eu acho que artesanato é uma riqueza, distrai. Desenvolve a criatividade, habilidade, então eu gosto muito. Ah, eu fiz muito casaquinho para os meus netinhos. Faço de vez em quando alguma coisa para dar de presente.* (P24G3, 18 meses de aposentadoria)

*Eu faço canto em um grupo para idosos* [...] *é uma coisa que gratifica muito. Preenche a alma da gente você cantar* [...] *eu crio letras de música* [...] *é uma coisa que me completa. Gosto muito de cantar* [...] (P14G2, três anos de aposentadoria)

Na aposentadoria, a maior disponibilidade de tempo possibilita que os indivíduos priorizem o autocuidado, fortaleçam vínculos sociais e familiares, adotem hábitos saudáveis e realizem atividades prazerosas que anteriormente eram inviáveis.

## DISCUSSÃO

Os achados deste estudo mostram que, embora a aposentadoria ainda seja significada como representativa da finitude do ser humano, ela também é significada positivamente como uma oportunidade para desfrutar melhor da vida. Destarte, os professores aposentados, consciente ou inconscientemente, com maior ou menor intensidade, vivenciam mudanças positivas em relação ao autocuidado, o que é resultante de um processo autorreflexivo e da interação social com familiares, profissionais de saúde, colegas de trabalho e pelo símbolo muitas vezes estigmatizado sobre o que é ser aposentado.

A partir dos resultados, foi possível perceber que a aposentadoria proporcionou/possibilitou dedicação de maior tempo às ações de autocuidado, além de maior autopercepção e conhecimento de si mesmo. Pesquisa realizada com 5.034 indivíduos holandeses, com idade entre 60 e 65 anos, identificou que a aposentadoria expõe as pessoas a novos contextos sociais, e isso pode levá-las a reavaliar suas orientações e formas de viver^(21)^.

Assim, por meio do processo de autoindicação, os aposentados determinam a sua linha de ação, o modo de vida que pretendem ter e o papel que irão assumir na sociedade. Ou seja, a autorreflexão é o mecanismo central pelo qual os aposentados passarão a enfrentar e lidar com o mundo.

Nesse sentido, o envelhecimento e a aposentadoria podem ser considerados como um dos objetos que, por meio do *self*, desencadeiam mudanças no autocuidado, como maior estímulo à memória, alimentação mais saudável, prática de exercícios físicos e consultas periódicas com profissionais de saúde. No caso de professores universitários aposentados, essas condutas podem ser decorrentes da interação social com pessoas que também têm mais acesso ao conhecimento sobre a importância dessas atitudes para a saúde, podendo tal temática estar envolta nas diferentes interações (*workshops*, congressos, participação em bancas, parecer *ad hoc*).

Ademais, o fato de a aposentadoria, mais frequentemente, ser relacionada ao envelhecimento e ter um símbolo estigmatizado, pode desencadear mudanças no comportamento dos aposentados, mediante maior envolvimento com o autocuidado. Desse modo, esperam que não sejam lembrados como aquela pessoa que perdeu sua utilidade para a família e/ou comunidade, não contribui com o desenvolvimento social e que só dá trabalho. A interação com outras pessoas possibilita a autorreflexão e, por conseguinte, o repensar e modificar, ou não, condutas, atitudes, comportamentos e planos de vida.

O desejo de, como aposentado, não ser visto de forma estigmatizada decorre da formação do objeto de si mesmo, mediante o processo de desempenho de papéis. A forma como o indivíduo se vê, como os outros o veem ou definem ou ainda a forma como a pessoa se vê assumindo um papel pode variar. Ou seja, durante a sua vivência, a pessoa pode ter tido interação com outros aposentados que adotaram como modelo a ser seguido ou negado, moldando a sua conduta^(13)^.

A importância da interação social para pessoas idosas foi reafirmada em estudo realizado com 12 participantes do grupo de convivência, os quais apontaram o ageísmo como fator que dificulta a inserção e interação social do idoso. Os dados evidenciaram também que a qualidade de vida da pessoa idosa está muito relacionada à alimentação saudável, prática de exercícios físicos, relações familiares, espiritualidade e religiosidade^(22)^.

A maior disposição para a realização de ações de autocuidado, o que muitas vezes é negligenciado durante o período de atuação profissional, para além de um processo de interação social e de interpretação, envolve também maior flexibilidade e disponibilidade de horários. Estudo qualitativo, realizado com 20 enfermeiros da rede básica e hospitalar, no estado do Rio de Janeiro, destacou o quanto o estilo de vida é comprometido e determinado pelo trabalho, isso porque, muitas vezes, os cuidados pessoais, como a vida social, os prazeres, as necessidades e as atividades direcionadas ao autocuidado, são negligenciados, devido a inúmeros compromissos laborais. O maior enfoque que muitas pessoas dão ao trabalho se relaciona ao *status* social e renumeração^(23)^.

A valorização e a maleabilidade de horários na aposentadoria também foram apontadas em uma revisão sistemática de estudos longitudinais. Neste estudo, foi possível identificar que a maior autonomia para administrar o tempo livre apresentou associação com aumento do tempo destinado à prática de esportes, lazer e atividade física^(17)^.

Nessa mesma direção, estudo realizado no Brasil, que utilizou como base o Estudo Longitudinal de Saúde dos Idosos Brasileiros, com uma amostra de 9.412 indivíduos com 50 anos ou mais, constatou que a maior disponibilidade de tempo possibilitava e incentivava o indivíduo a investir mais em sua saúde. Inclusive, leva-o a perceber-se como sendo menos laborioso ir a uma consulta médica ou cozinhar alimentos mais saudáveis^(24)^.

Estudo realizado no Japão, com dados obtidos do *Japan Household Panel Survey* com indivíduos de 50 anos ou mais, aposentados e não aposentados, constatou que, em média, os aposentados estavam mais satisfeitos em relação às atividades de lazer, saúde e vida do que os não aposentados. Ademais, a aposentadoria foi associada ao bem-estar psicológico, obtendo um declínio nos sintomas psicossomáticos, somáticos, sofrimento psicológico, com melhorias na satisfação com a vida, bem como aumentos nos hábitos de exercício físico^(25)^.

No Brasil, dados da Pesquisa Nacional de Amostra de Domicílio apontam que a aposentadoria possibilitou maior disponibilidade aos familiares ao lazer, cuidados com a saúde e bem-estar, propiciando benefícios para a vida pessoal e familiar, resultando em diminuição na ocorrência de diversas doenças e nos indicadores de saúde como um todo. Ou seja, a aposentadoria proporciona mais tempo para o cuidado de si e dos que fazem parte do seu cotidiano^(26)^.

Os benefícios do autocuidado com a saúde na aposentadoria são decorrentes de maior disponibilidade de tempo para o lazer, maior flexibilidade de horário e da ausência de compromissos diários. Pesquisa realizada com 5.405 americanos com 50 anos ou mais constatou que atividades como leitura, artesanato e música tendem a estimular e ativar a memória, atenuar a associação negativa entre aposentadoria e declínio da função cognitiva e dos sintomas depressivos^(27)^.

Nesse contexto, pesquisa sueca investigou a associação longitudinal entre atividade de lazer e sintomas depressivos nos primeiros três anos de aposentadoria em 1.033 aposentados, constatando que aqueles com sintomas depressivos intensos no início do estudo tiveram reduções lineares no envolvimento em atividades sociais/lazer. Já os indivíduos que tinham maior envolvimento em atividades sociais e físicas no início da aposentadoria mencionaram redução da sintomatologia depressiva^(28)^. Por isso, destaca-se a importância do processo de preparação para a aposentadoria, de modo que as pessoas possam desenvolver estratégias que amenizem as repercussões na saúde mental. De qualquer forma, os profissionais de saúde precisam estar atentos para identificar precocemente sinais depressivos e realizar a assistência à saúde de acordo com as necessidades apresentadas, considerando a integralidade do cuidado.

Vale lembrar que o déficit de autocuidado em pessoas idosas está relacionado a maior prevalência de comorbidades, à deterioração na percepção subjetiva da saúde, à crescente dependência para a realização das atividades da vida diária e maior propensão à inatividade física regular, o que exacerba o declínio funcional. Dessa forma, é substancial o desenvolvimento de intervenções estratégicas e direcionadas que considerem as especificidades do envelhecimento e promovam a integração dos indivíduos às políticas públicas e aos recursos comunitários. Essas ações visam fomentar a autonomia e fortalecer a prática do autocuidado, contribuindo para a melhoria da qualidade de vida e para a redução dos impactos negativos sobre o sistema de saúde^(29)^.

Destarte, o autocuidado desempenha papel crucial na manutenção da saúde física, emocional, mental, espiritual e social dos indivíduos. Avaliar regularmente as necessidades em cada uma das áreas com identificação de padrões disfuncionais de autocuidado e obstáculos à sua implementação possibilita uma abordagem mais individualizada, favorecendo a adoção de práticas e comportamentos adequados e conscientes^(30)^.

Frisa-se que as atividades desenvolvidas na aposentadoria são moldadas por percepções positivas ou negativas sobre o processo de envelhecer. Nessa direção, pesquisa realizada com 24 atletas de alto rendimento aposentados há mais de quatro anos observou que aqueles com ideias positivas sobre o envelhecimento se sentiam motivados à prática de exercícios físicos, enquanto os que tinham uma percepção negativa, marcada por medos, inseguranças e estigmas, associavam a aposentadoria atlética a perdas. A preparação para a aposentadoria e a escolha do momento em que a mesma deveria ocorrer influenciaram a sua aceitação e, consequentemente, os comportamentos após esse evento. Ademais, a interação com familiares demostrou ter importância ímpar, ao incentivar e orientar práticas de autocuidado, como, por exemplo, a de se manter fisicamente ativo^(31)^.

O papel da interação com familiares também foi destacado em estudo realizado na China, o qual constatou a importância do companheirismo e dos cuidados dos cônjuges na manutenção do estilo de vida saudável, sobretudo para idosos acima de 70 anos. Constatou-se também que o impacto negativo de viver sozinho nos hábitos alimentares foi maior para as mulheres do que para os homens, provavelmente devido às diferenças de *status* social e responsabilidades familiares. Qanto menos identidade social a pessoa tem, mais impacto negativo sofrerá quando perder uma delas e, após a aposentadoria, os homens tendem a manter muitas identidades sociais, enquanto as mulheres tendem a assumir majoritariamente o papel de dona de casa^(32)^.

Nesse tocante, estudo realizado na Alemanha com 11.168 pessoas na faixa etária de 45 a 80 anos destaca que os profissionais de saúde devem considerar, de forma holística, os fatores comportamentais e as circunstâncias em que as pessoas aposentadas estão inseridas. Não adianta apenas incentivar a prática de exercícios físicos e lazer, devido a essa maior flexibilidade de horários na aposentadoria e senescência, mas deve-se estabelecer uma parceria e elaborar conjuntamente (profissional e aposentado) um plano de cuidados que seja individualizado, considerando possíveis dificultadores para o exercício, tais como problemas articulares, fraqueza muscular, disponibilidade de recursos estruturais e sociais para esta atividade, entre outros^(33)^.

Outro aspecto a ser considerado é que os cuidados com a aparência estão relacionados às regras existentes no meio social, podendo ser reflexo de interações sociais, liberdade, autoconhecimento, autodeterminação e reconhecimento^(34)^. No entanto, tais cuidados nem sempre proporcionam benefícios para a saúde mental e física dos indivíduos, isso porque os mesmos podem ser realizados apenas para evitar estigmas decorrentes dos símbolos negativos vinculados ao envelhecer e não por um desejo pessoal expresso. Nesse sentido, reitera-se que a realização de atividades de autocuidado motivada apenas para evitar estigmas não é benéfica.

É fundamental compreender como ocorre a interação social do aposentado com o mundo e com o seu *self,* pois, às vezes, a pessoa até desenvolve ações de autocuidado de forma satisfatória, mas internamente essas ações podem ter mais relação ao estigma do símbolo de ser aposentado, não ao desejo de desfrutar inteiramente dos benefícios que a aposentadoria pode desencadear. Por isso, são tão divergentes os resultados de benefícios e malefícios da aposentadoria na vida das pessoas^(35)^.

Destarte, fazem-se necessárias estratégias que possibilitem que a aposentadoria ocorra de forma personalizada e satisfatória. Sugere-se que as pessoas se preparem para a aposentadoria, participando de intervenções com abordagens interdisciplinares, mediante a realização de consultas com profissionais especializados de diversas áreas, palestras instrucionais e reuniões em grupo^(2,36)^.

### Limitações do estudo

Possíveis limitações do estudo podem estar relacionadas ao fato de as entrevistas terem sido realizadas de modo remoto devido às restrições impostas pela pandemia de COVID-19. Isso pode ter impossibilitado que pessoas com dificuldade em acessar a rede remota participassem do estudo. Ademais, os participantes terem sido incluídos a partir da indicação de pares e da disposição em compartilhar suas experiências após a aposentadoria pode ter circunscrito os resultados.

Contudo, apesar do risco teórico de viés devido à predominância de uma área específica (enfermagem), os dados empíricos não mostraram impacto considerável dessa diferença, pois o desejo ou mesmo necessidade de mudanças também foi percebida por profissionais da área da saúde, demonstrando que a formação e a prática profissional não são suficientes para garantir a adoção de estilos de vida mais saudáveis antes da aposentadoria, o que fortalece a confiabilidade do estudo.

### Contribuições para a área da saúde e enfermagem

Os resultados do estudo trazem contribuições importantes para maior compreensão sobre as implicações da aposentadoria sobre o estilo de vida dos aposentados. Destarte, compreender, a partir da lente dos próprios aposentados, os significados envolvidos na adoção de novos comportamentos relacionados à saúde e que resultam em medidas de autocuidado potencializa as possibilidades de planejar ações, no âmbito individual e coletivo, voltadas para a melhoria das condições de vida e saúde dos aposentados.

Os resultados destacam também a importância de os profissionais de saúde valorizarem as características das diferentes etapas do ciclo vital do ser humano, reconhecendo que aqueles em pré-aposentadoria e também os aposentados necessitam de um olhar ampliado, de modo que possam constituir fonte de apoio e estímulo às mudanças comportamentais, sobretudo aquelas relacionadas ao bem estar e à saúde nesta importante etapa da vida.

## CONSIDERAÇÕES FINAIS

Identificou-se que a experiência da pessoa durante a aposentadoria possui dois pólos. Inicialmente, há uma maior propensão a que a pessoa sofra com o medo da estigmatização que o aposentar-se traz para o convívio e a interpretação social. Uma vez superada essa dificuldade inicial, os aposentados passam a reconhecer que há benefícios que anteriormente não podiam ser experienciados, decorrentes principalmente de maior disponibilidade e maleabilidade para a gestão do tempo. Isso, por sua vez, os impulsiona a vislumbrar possibilidades de adoção de práticas mais consistentes e constantes de autocuidado. Há uma maior dedicação de tempo à prática de *hobbies* e exercícios físicos, além de cuidado com a aparência, memória, alimentação e vida social. Isso ocorre por influência das interações sociais (com amigos, familiares, profissionais de saúde, colegas de trabalho) ao longo da vida e, nesta etapa em particular, devido à maior disponibilidade e flexibilidade de horários e em decorrência do próprio processo de envelhecimento.
